# Development and Characterization of Non-Evaporable Getter Thin Films with Ru Seeding Layer for MEMS Applications

**DOI:** 10.3390/mi9100490

**Published:** 2018-09-25

**Authors:** El-Mostafa Bourim, Hee Yeoun Kim, Nak-Kwan Chung

**Affiliations:** 1 National Nanofab Center, Department of Nanostructure Technology, Daejeon 34141, Korea; hyeounkim@nnfc.re.kr; 2 Korea Research Institute of Standard and science, Center of Vacuum and Technology, Daejeon 305-340, Korea; nk.chung@kriss.re.kr

**Keywords:** non-evaporable getter, non-evaporable getter (NEG), activation temperature, sorption properties, sorption speed, sorption capacity

## Abstract

Mastering non-evaporable getter (NEG) thin films by elucidating their activation mechanisms and predicting their sorption performances will contribute to facilitating their integration into micro-electro-mechanical systems (MEMS). For this aim, thin film based getters structured in single and multi-metallic layered configurations deposited on silicon substrates such as Ti/Si, Ti/Ru/Si, and Zr/Ti/Ru/Si were investigated. Multilayered NEGs with an inserted Ru seed sub-layer exhibited a lower temperature in priming the activation process and a higher sorption performance compared to the unseeded single Ti/Si NEG. To reveal the gettering processes and mechanisms in the investigated getter structures, thermal activation effect on the getter surface chemical state change was analyzed with in-situ temperature XPS measurements, getter sorption behavior was measured by static pressure method, and getter dynamic sorption performance characteristics was measured by standard conductance (ASTM F798–97) method. The correlation between these measurements allowed elucidating residual gas trapping mechanism and prediction of sorption efficiency based on the getter surface poisoning. The gettering properties were found to be directly dependent on the different changes of the getter surface chemical state generated by the activation process. Thus, it was demonstrated that the improved sorption properties, obtained with Ru sub-layer based multi-layered NEGs, were related to a gettering process mechanism controlled simultaneously by gas adsorption and diffusion effects, contrarily to the single layer Ti/Si NEG structure in which the gettering behavior was controlled sequentially by surface gas adsorption until reaching saturation followed then by bulk diffusion controlled gas sorption process.

## 1. Introduction

Non-evaporable getters (NEGs), based on the thin film deposition technique, were until now still the practical technical solution for integrating a scaled-down getter into microelectronic devices, that need special residual gas control within their cavities for keeping their functioning performances optimal after packaging under a controlled atmosphere. Among these devices, micro-electro-mechanical systems (MEMS) are of high utility for scalable NEGs. Depending on their global functions, MEMS can be exploited to work either under vacuum or some controlled residual gas pressure of an inert gas inside their cavities [1,2]. Unfortunately, degradation of the desired pressure atmosphere inside the MEMS devices is commonly primed by effects such as surface degassing, micro-leaks as well as permeation. Thus, a very slight leak would cause significant variations in the adjusted internal atmosphere pressure into the MEMS cavities and hence alter their assigned operation. Therefore, the ability of the getter to trap these residual and/or additional gases can be exploited to efficiently master the vacuum or atmospheric pressure conditions into the MEMS cavity that assures their optimal performance and reliability throughout the system lifetime [2,3]. As known, getter materials are chemical pumps able to absorb active gases (H_2_, CO, CO_2_, O_2_, N_2_, H_2_O, etc.) on their surfaces and the most common basic metals for NEGs are Ti and Zr either in their single phase states or as alloys composed of other metallic elements in intermetallic compounds [4,5,6,7]. Ti and Zr belong to the group V elements in periodic table possessing a high oxygen solubility limit and high oxygen diffusivity physical properties, which are behind the high gas sorption properties (sorption speed and capacity) manifested by NEGs. In fact, the NEGs gettering performances are tightly linked to the NEGs fabricated microstructure, which is required to be essentially formed of a small columnar grain structure that promotes the gas sorption process by diffusion through a NEG grain boundary microstructure. NEGs are operational only after their activation under vacuum at a high temperature. However, the getter should be developed for low activation temperatures compatible with the sealing process temperature of MEMS devices. Usually, the most well-known bonding processes for MEMS cavity hermetic sealing their processing temperature are performed at temperatures ranging from 300 °C to 450 °C [8]. Therefore, a getter with an activation temperature less than 450 °C would be considered as a solution to the issue that MEMS device can withstand during the sealing process.

To optimize sorption performances and reduce thermal activation temperature of NEGs systems, in the literature, many investigations have been undertaken to improve the NEG materials diffusivity by fabricating them from alloys of several mixed metals [5,9,10,11,12] or by controlling the NEG microstructure and surface roughness using pure multi-metallic-layer thin films forming NEGs in a stacked structure [13,14,15]. The investigation of getter formed of metal alloys needs a minute analysis of each metallic element involved in the alloy composition, which makes it certainly not easy to elucidate precisely the mechanism controlling the gettering process. 

In this work, we have chosen to investigate the getters formed of pure metallic thin films stacked in single and multi-layered structures such as Ti/Si, Ti/Ru/Si, and Zr/Ti/Ru/Si. The insertion of the ruthenium as a seed sub-layer, on which the elementary getter film layers are deposited, is for growing the getter microstructure in small columnar grains with upper surface morphology manifesting a high roughness. The all metallic films composing the getter are deposited under the same temperature and pressure conditions, and the finer crystalline microstructure growth depends on the highest melting point. Therefore, as the ruthenium metal possesses a higher melting point, its film layer growth will therefore form in a thinner columnar microstructure defined by a higher grain boundary density [16,17]. Thus, the growth of the subsequent getter film layer is partially controlled by a limited mobility of its metal atoms at the Ru sub-layer surface thereby leading to a finer microstructure in the getter film, consisting of a columnar grain structure and a high getter surface roughness. Such a microstructure promotes the getter to be activated at a low temperature due to the diffusion process mostly taken along the grain boundaries, as well as provides the getter with a large sorption capacity, sufficient for pumping throughout the MEMS device lifetime.

Therefore, to confirm the Ru seed sub-layer effect on getter microstructure control, activation temperature and sorption characteristics, in this study, we compared the microstructure and morphology of the getters fabricated with and without a Ru seed sub-layer. In addition, we analyzed the in situ temperature XPS getter surface chemical state modifications after thermal activation and we measured the N_2_ sorption behavior in the pressure–time dependence and sorption performance properties after the activation process at different temperatures. A correlation relating the XPS getter surface chemical state changes to the getter sorption behavior was evidenced, and hence the getter activation and the corresponding gas sorption mechanisms were elucidated.

## 2. Experimental Section

NEG thin films were fabricated by deposition on 4” (100) Si substrates using an e-beam and thermal evaporation system (KVE & T-C500200, Korea Vacuum Tech, Ltd., Gimpo-si, Korea). The substrates were prepared in accordance with the standard RCA cleaning method. Depositions were performed at room temperature and at a base pressure of 5 × 10^−8^ Torr, the vacuum environment avoids getter contamination by the carrier gas as used in the deposition by the sputtering technique. Generally, such deposition conditions promote nano-grain formation in a columnar-like structure basically controlled by the reduced atomic mobility of the deposited species on the substrate and self-shadowing during the film growth [16,17]. The evaporated materials used were titanium (99.9995% stated purity), zirconium (99.9995% stated purity) and ruthenium (99.95% stated purity). The deposition rate was 1 nm/s for the Ti and Zr layers and 0.5 nm/s for the Ru one. The average thicknesses of the deposited films were 30 nm for the Ti adhesive layer, 600 nm for the Ti layer in the getter structure with a single active film, 300 nm for each of the Ti and Zr layers in the getter structure with double active films, and 60 nm for the Ru seed sub-layer. The surface morphology and the fracture cross-sectional structure of the fabricated NEGs were analyzed by field emission scanning electron microscopy (FE-SEM, S-4800, Hitachi, Tokyo, Japan) system. An atomic force microscope (AFM, SPI4000, Seiko instruments, Chiba, Japan) was used to determine the surface roughness. In situ temperature X-ray photoelectron spectroscopy (XPS) analyses were recorded using an ESCALAB 250 (Thermo Fisher Scientific, Loughborough, UK) XPS spectrometer equipped with a monochromatic Al Kα X-ray source and a ceramic heater stage. The NEG sample was heated by direct contact with the ceramic heater and the temperature was controlled with a temperature controller that covered a temperature range from RT to 600 °C. Before each temperature XPS measurement, the NEG sample was placed in an ultra-high vacuum (UHV) chamber and maintained for one hour at the targeted temperature before the XPS data were recorded. For consistency, the chamber pressure was maintained below 1 × 10^−7^ Torr during the measurements, regardless of the NEG sample temperature.

As the film surface of the fabricated getter was exposed to the air during its transfer, it was easy to oxidize and to be contaminated with others gases. Thus, a passive layer was formed on the getter surface. This passivation layer was a protective barrier against more gas absorption and diffusion, resulting in gettering deterioration. It was necessary, before testing the gettering property performance, to regain the getter active surface by annealing the getter sample under a high vacuum and a high temperature, this dissolves and diffuses the protective oxide surface into the getter film bulk. Such operation, leading to a fresh getter surface, is called an activation process. For an effective activation, the getter material must also have the ability to allow diffusion of the contaminating gases once they have been absorbed. 

**Figure 1:**
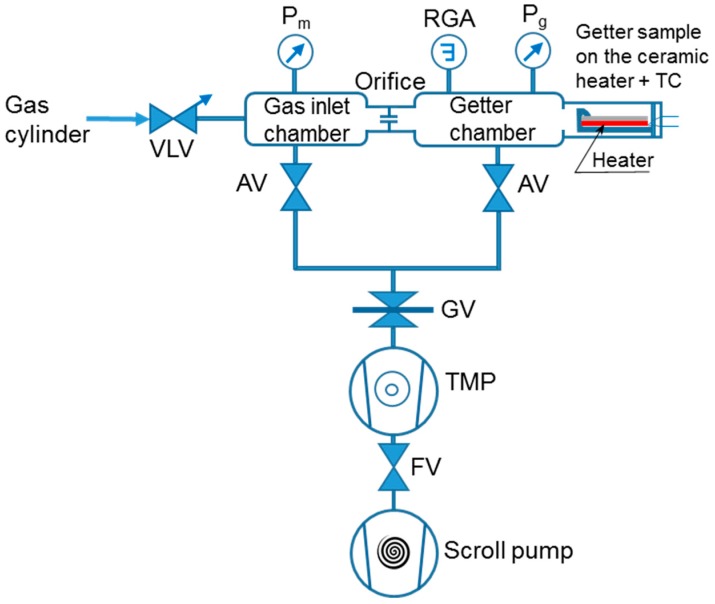
Schematic diagram of the getter test system. *P_m_*, *P_g_*: Pressure gauge, AV: Angle valve, GV: Gate valve, VLV: Variable leak valve, TMP: Turbo molecular pump, FV: Fore-line valve, RGA: Residual gas analyzer (device dismantled for total pressure measurements).

For characterizing the activation effect on the getter gas sorption behavior and for evaluating the getter sorption property performances, a getter test system was used (Figure 1). This experimental system was designed based on the standard ASTM-F798 method [18] for the evaluation of gas sorption characteristics. The system consisted of two communicating chambers (getter chamber and gas inlet chamber), through a conductance orifice. These chambers could be simultaneously evacuated or isolated by actuating manually the vacuum valves (AV; EV-150, Huntington Mechanical Labs, Inc., Grass Valley, CA, USA and GV; E-GV-2500M, MDC Vacuum Products, LLC, Hayward, CA, USA) located on the bypass passages connected to the pumping units (Turbo-molecular pump (TMP) and Scroll pump). The getter chamber was made in a cylindrical tube form (diameter = 35 mm and length = 150 mm) from stainless steel, as the rest of all vacuum system parts, and the measured getter sample installed inside was of a rectangular shape with gettering surface area dimensions of 2 cm × 6 cm. The pressure in each chamber was measured using a pressure gauge (*P_g_*, *P_m_*) Bayard Alpert type hot cathode ion gauges (PBR 260, Pfeiffer Vacuum, Asslar, Germany). Nitrogen (N_2_) gas was admitted in the inlet chamber via a UHV high-precision variable leak valve (VLV; ULV–150, MDC Vacuum Products, LLC). The total system was evacuated by a 60 L/s TMP (TMU 071P, Pfeiffer Vacuum) in series with an oil-free dry scroll vacuum pump (ISP–90 Scroll Meister, Anest Iwata, Inc., Cincinnati, OH, USA). The ceramic heater was made from an alumina plate with a smooth surface finish with a resistance heating element of 50 Ω at RT; it could reach a maximum temperature of 500 °C (CMTECH; Daegu, Korea). A K–type thermocouple (TC) inside the getter chamber and in contact with the heater was used for measuring the temperature of the getter. Regarding the residual gas analyzer (RGA) element, figuring on the getter test system schematic diagram, it is worth to mention that this device was not installed on the system in the experiments for measuring the effective total pressure in the chambers. Such action was considered in order to avoid any parasitic effect on the measured pressures, leading to ambiguous results and difficulties in interpretations.

To ensure comparability of the sorption tests between the samples, once the getter chamber was opened to the atmosphere, a conditioning assuring the same initial getter chamber pressure before starting the pressure measurements was performed. The getter sample was attached directly to the heater, and both were mounted on a holder isolated from the chamber walls; such set-up focused the heat locally on the sample only. Once the sample was placed in the getter chamber, all the getter test system was backed out at a temperature of 130 °C for 48 h in order to clean the chambers internal walls that minimized the vacuum chamber outgassing and also allowed the getter chamber to reach an UHV of 10^−8^ Torr.

Regarding the investigations of the activation effect on the pressure–time dependence, the measurements were performed by means of a static method. In this method, after activating the getter sample at a specific temperature for one hour, and subsequent cooling back down to room temperature, the all valves (AV and GV) of the getter test system were closed to isolate the getter chamber from the pumping line (TMP and scroll pump), and then immediately after a moment of time of about 3 min, when the getter chamber pressure had stabilized around 10^−7^ Torr, a flow of pure nitrogen (N_2_) gas was induced through the high-precision variable leak valve (VLV) to rapidly reach a pressure of 10^−5^ Torr inside the getter chamber. The gas was immediately stopped, by closing the leak valve, once the set pressure has been reached. Pressure–time dependence was recorded for a period of about 3 h, starting from the moment of gas-inlet valve closing until the chamber outgassing pressure became largely dominant.

Regarding the measurements of the sorption properties (sorption speed and capacity), a standard called the dynamic method was used. It is described by the standard conductance technique in ASTM–F798 [18], and its basic principle is based on the measurement of the pressure difference between two chambers; the gas inlet chamber (*P_m_*) and the sample chamber (*P_g_*) (see schematic drawing of the setup in Figure 1). These chambers are connected to each other through a small orifice. The sorption speed of a getter was calculated as follows:

(1) $$S=\frac{Q}{P_{g}}\;\mathrm{and}\;Q=C_{0}\left(P_{m}-P_{g}\right).$$

Here, *S* is the sorption speed of the getter (L/s·cm^2^), *Q* is the sorption capacity (mbar·L/cm^2^), *C*_0_ is the conductance orifice with diameter = 2 mm and C = 0.4 L/s. 

In this method, after the getter was activated and the pressure reached the UHV, around 10^−8^ Torr (in both gas inlet and getter chambers) at room temperature, all the valves (AV and GV) were closed and after a few minutes (about 3 to 5 min) of pressure stabilization around 10^−7^ Torr (in both gas inlet and getter chambers), the nitrogen gas was admitted in the gas inlet chamber through the high-precision variable leak valve (VLV) manually tuned to let a weak flow, maintaining a constant pressure of 10^−5^ Torr, into the gas inlet chamber. The pressures into the two chambers were recorded with time until they equalized when the getter surface became saturated.

## 3. Results and Discussion

### 3.1. Microstructure and Morphology Analyses

**Figure 2:**
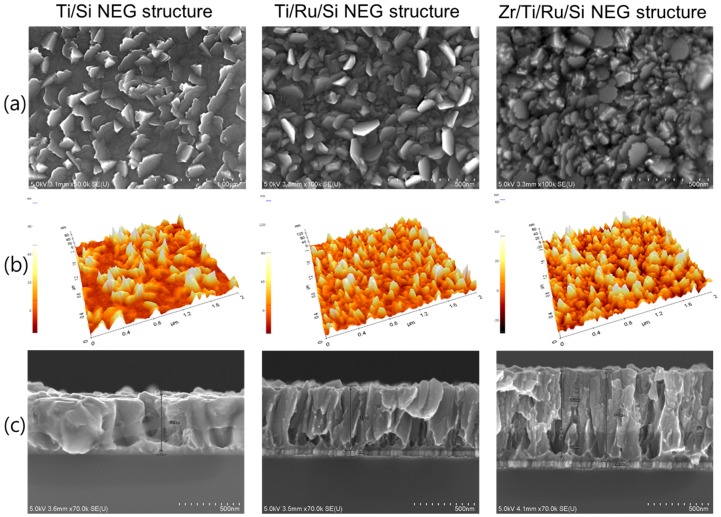
SEM surface micrographs (**a**), 3–D AFM images (**b**), and SEM fracture cross-section micrographs (**c**) of single Ti/Si NEG (left column); multilayer Ti/Ru/Si NEG (middle column) and multilayer Zr/Ti/Ru/Si NEG (right column).

Microstructure analysis by SEM and AFM observations for the three fabricated NEGs are shown in Figure 2. Generally, the images show partially entangled leaflets (small leaves) grown vertically on each getter sample surface. These leaflets were stacked almost vertically with their normal plane orientated in different directions, which allow an inter-granular arrangement with cavities lying down from the surface to the film bulk. Furthermore, multilayered NEGs structures with a Ru sub-layer show a dense leaflet surface morphology compared to the single layer Ti/Si NEG, whose surface was a mixture of an inhomogeneous distribution of embedded leaflets and flat grain clusters. Given the complex morphology showed by all the NEG surfaces, it was quite difficult to give a representative grain size value from the NEG upper surface.

Therefore, from a combination of the different information gained by SEM observations and AFM roughness measurements (Figure 2a,b), an average estimation of the leaflet width and surface roughness is given in Table 1. In addition, an important insight into microstructure growth was obtained from the cross-sectional SEM observation of the fracture surfaces (see Figure 2c). For the NEG samples without a Ru sub-layer, it was observed that at the Si*_substrate_*/Ti interface there was a dense compact Ti thin layer with a variable thickness of a few tens-of-nm all along the sample cross-section. This was just an indication that at the beginning of Ti film deposition, the early growth of the film had developed, on the Si substrate, a nano-undulated-patterned thin layer composed of hills and craters with a wide distance between the hill summits. From this type of hills and craters pattern, it looked as though the nucleation and growth of large columnar grains were taking place from the craters and thin leaflets were taking place from the hill summits. Thus, the Ti microstructure had been raised up. Regarding the Ti/Ru/Si and Zr/Ti/Ru/Si NEG samples, the deposited Ru sub-layer (~60 nm) had shown a very narrow and long columnar structure, which had a significant influence on the surface interface for controlling the subsequent compact layer of the early deposited Ti film to be manifested in a microstructure with a high nano-undulated density pattern. Hence, from the resulting small craters, with very close hill summits at the beginning of Ti layer growth, a very well-defined Ti columnar structure formed only in a leaflet shape had been nucleated and grown up. For the Zr/Ti/Ru/Si NEG sample, the already deposited Ti film, conditioned in leafleted microstructures by Ru sub-layer, has served as a seed layer for Zr film growth, and thus the Zr film microstructure has manifested once again only in a well-defined leaflet columnar structure, but with very small size and random direction orientation of leaflet faces.

From the above analysis, it turned out that the multilayer thin film based getters using Ru as a sub-layer had a film microstructure composed of inclined columnar leaflets that were finely packed as nano-crystal columns with different levels emerging from the getter surface. Such microstructure gives rise to a high density of grain boundaries, a high porosity and a high surface roughness with a high specific area. High roughness and porosity promote a surface with, both, a high gas capacity and a high gas sticking probability, while the high grain boundary density promotes a high gas bulk diffusion. All of these features are requested for a practical NEG to present high sorption properties and performance.

### 3.2. In-Situ XPS Getter Surface Analysis during Thermal Activation

**Figure 3:**
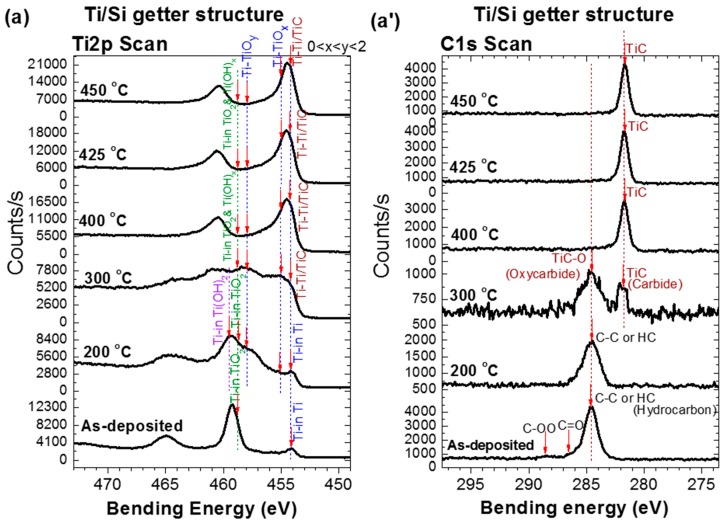

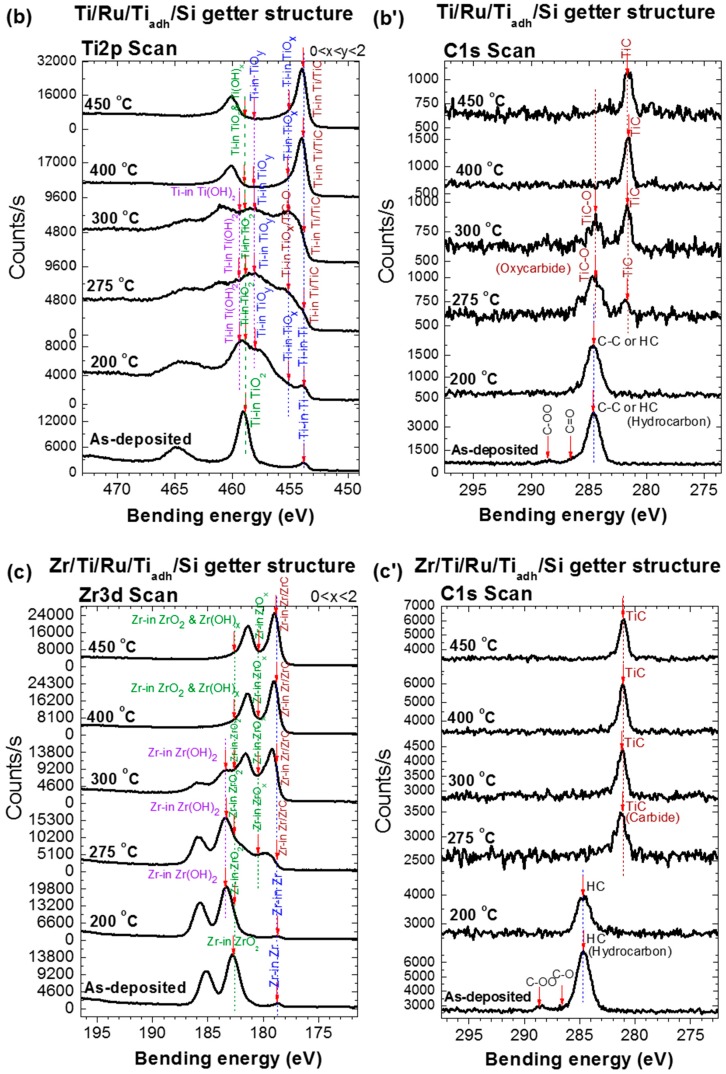
Evolution of XPS spectra after activation at various temperatures. (**a**,**a’**) Ti 2p and C 1s orbitals for the single layer Ti/Si NEG; (**b**,**b’**) Ti 2p and C 1s orbitals for the multilayer Ti/Ru/Si NEG; (**c**,**c’**) Zr 3d and C 1s orbitals for the multilayer Zr/Ti/Ru/Si NEG.

The surfaces of the fabricated getters were investigated by in situ temperature XPS under the same conditions (annealing under UHV for one hour) as during their activation in the getter test system. Figure 3 shows the in situ XPS surface characteristics of the three studied NEG samples. The measured data are related to the evolution of the XPS spectra of Ti 2p, Zr 3d and C 1s orbitals during the activation process at various temperatures. The analysis of theses XPS characteristics allowed information about the getter upper surface chemical state modifications to be obtained as well as the reactions of the surface contaminants (O, C, H, etc.) with the main metals (Ti or Zr) of each NEG upper layer. Indeed, the reduction of the oxides of these elements to their metallic states is a clear indication of NEG activation by demonstrating a clean reactive surface [19,20,21]. Furthermore, knowing how the contaminants bound to the getter metal may provide insight into understanding the gettering mechanism and performance. In the following, in the XPS analysis, since the evolution trends of XPS oxidation states with temperature for Ti upper-layer NEG are almost similar to the Zr upper-layer NEG; therefore, to avoid repetition in the explanation, when describing the chemical state change undergone by the titanium, the corresponding zirconium state transformation is mentioned in parentheses. 

As shown in Figure 3a–c, the evolution with temperature of the XPS spectra associated with the Ti 2p orbital of the Ti upper-layer NEGs and the one associated with the Zr 3d orbital of the Zr upper-layer NEG have two regimes. In the first regime from the as-deposited NEG sample to the activation at 275 °C, the Ti 2p (Zr 3d) spectrum evolution was mainly controlled by the decrease of the doublet peak Ti^4+^ (Zr^4+^) of bending energy (BE): $\mathrm{Ti}\;2p_{3/2}$ = 458.8 eV, $\mathrm{Zr}\;3d_{5/2}$ = 182.8 eV related to the TiO_2_ (ZrO_2_) oxide, accompanied simultaneously with, both, an increase in the Ti^x+<4^ (Zr^x+<4^) doublet peaks related to TiO_x<2_ (ZrO_x<2_) suboxides [13,21,22,23,24,25,26,27] and an emergence of a metal hydroxide Ti(OH)_2_ (Zr(OH)_2_) doublet peak located at higher bending energies ($\mathrm{Ti}\;2p_{3/2}$ = 459.3 eV ($\mathrm{Zr}\;3d_{5/2}$ = 183.4 eV)) [28,29,30,31]. In the second regime, over 275 °C and up to 450 °C, the Ti 2p (Zr 3d) spectrum evolution was mainly controlled by a simultaneous decrease of metal-oxide, metal-hydroxide, and metal-suboxide, accompanied with a significant increase of doublet peak related to the metallic state Ti^0^ (Zr^0^) with a BE of ${\mathrm{Ti}}^{0}\;2p_{3/2}$ = 454 ± 0.2 eV (BE of ${\mathrm{Zr}}^{0}\;3d_{5/2}$ = 178.9 eV) as well as some contaminant-based phases peaks such as carbides TiC, TiC–O (ZrC, ZrC–O) [13,21,22,23,24,25,26,27]. Thus, it seems that the Ti 2p (Zr 3d) spectrum has gradually moved towards the right, towards lower binding energies by comparison with the initial spectrum position of the oxidized as-deposited NEG. Otherwise, this implies that the initial oxide surface layer of the as-deposited getter was gradually reduced to a metallic state with increasing thermal activation temperatures [13,21,22,23,24,25,26,27].

It is to be noted that the metal-hydroxide (Ti(OH)_2_, Zr(OH)_2_) XPS doublet peaks appeared after activating at 200 °C (see peak indexations on Figure 3a–c). For the Ti upper-layer of Ti/Si and Ti/Ru/Si NEGs, the $\mathrm{Ti}\;2p_{3/2}$ peak maximum level was reached also at 200 °C, with almost a small shift of the Ti 2p spectrum to a higher bending energy (BE = 459.4 eV) [28,29]. Whereas for the Zr upper-layer of the multilayered Zr/Ti/Ru/Si NEG, the $\mathrm{Zr}\;3d_{5/2}$ peak maximum level was reached at an activation temperature of 275 °C, with a substantial shift of the Zr 3d spectrum towards a higher BE = 183.4 eV [27,30,31].

The C 1s XPS spectrum evolution with thermal activation for all three NEGs also showed two different regimes (Figure 3a’–c’) [13,21,22,23,24,25,26,27]. In the first regime, for the Ti upper-layer of the Ti/Si and Ti/Ru/Si NEGs, the main elementary surface-adsorbed C–C or C–H peak (BE = 285.3 eV) underwent a transformation to a metal-oxycarbide (TiC–O), accompanied with a metal-carbide (TiC) at an activation temperature of 300 °C. While for the Zr upper-layer of the multilayered Zr/Ti/Ru/Si NEG, only one direct transformation to a metal-carbide (ZrC) at 275 °C was observed. In the second regime, the only persisting peak of the carbide phase (BE = 281.6 eV) showed a slight intensity increase up to 450 °C for multilayered NEGs with a Ru sub-layer than for a single layer Ti NEG. 

For more additional details, the origin of the metal-hydroxide, carbide and oxycarbide bonds results probably from the dissociation of water molecules, O–H and C–H groups already adsorbed on the as-deposited NEG surface, or from vacuum chamber contaminants. It is important to note that the metal-hydroxide bond formation manifested at the first stage of heating, and its maximal concentration was accompanied by oxide reduction on the film surface as confirmed to happen around 250 °C in many reports in the literature [27,30,32]. As the hydroxide group elements were randomly distributed on the getter surface, M–HO_x_ chemical-bond clusters formed through the hydrogen dissociation and migration into the getter sub-surface would generate an inhomogeneous oxygen-depleted region all around these clusters on the surface with different Ti^x+,y+<4^ (Zr^x+,y+<4^) low oxidation states [27,30,32]. 

Regarding carbide/oxycarbide formation, the oxide reduction leading to metallic and suboxide states was a necessary condition. Thus, the temperature at which the carbide/oxycarbide starts to be formed correlates well with the metallic Ti^0^ (Zr^0^) and suboxide (Ti^2+,3+^, Zr^1+,2+^) state appearance [13,21,22,23,24,25,26,27]. Furthermore, the characteristic energy of these carbon-based compounds on the Ti 2p (Zr 3d) spectrum was very close to their corresponding metallic and suboxide states; such as the TiC (ZrC) bending energy superposes on that of the Ti^0^ (Zr^0^) metallic state, and the one for TiC–O (ZrC–O) superposes on that of Ti^x,y<4^ (Zr^x,y<4^) in TiO_x,y<2_ (ZrO_x,y<2_) suboxides [25,27].

**Figure 4:**
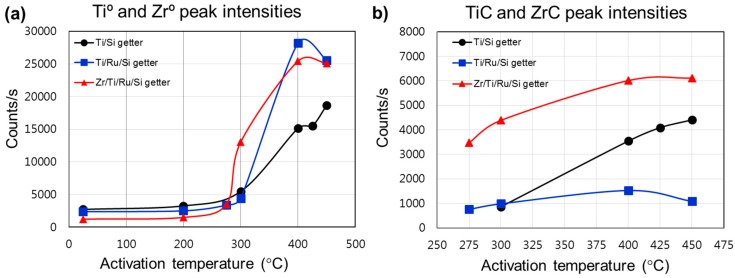
Plots of XPS elemental intensities as a function of activation temperature. (**a**) For Ti^0^ and Zr^0^ metallic states; (**b**) for TiC and ZrC carbide compounds.

In order to establish a comparison elucidating the performance related to each investigated getter structure, we plotted (Figure 4) the intensities of the metallic and carbidic states evolution during the thermal activation. The metallic states generation on the getter surface enhanced the sorption properties, while the formation of carbide or other stable compounds reduced the getter activity by reducing the density of available metallic adsorption sites favorable to react with gas species on the surface. Hence, the distribution and evolution of the metallic and carbidic species on the getter surface gave insight into the gettering mechanism and performance evaluation after each activation process. Figure 4a shows the comparison between the metallic state intensity variation and activation temperature. The metallic state (Ti^0^, Zr^0^) intensity was shown to be higher for getters with a structure using a Ru sub-layer, whereas the Zr/Ti/Ru/Si NEG presented better values compared to the Ti/Ru/Si NEG before activation at 400 °C. Furthermore, it was also seen that the activation of the Zr/Ti/Ru/Si NEG starts to be triggered at a temperature lower than the other getters. Meanwhile, the carbidic state intensity was found to be higher for the Zr/Ti/Ru/Si NEG compared to Ti-upper layer based NEGs without and with a Ru sub-layer (Figure 4b). Also to be noticed is that the carbidic intensity level, for the getters of a structure with a Ru sub-layer, underwent an apparent continuous increase with activation temperature from its appearance in the second regime.

From the above analysis, two key conclusions can be drawn. Firstly, the metal-hydroxide (Ti(OH)_2_, Zr(OH)_2_) formation, which induces a high getter sub-surface reduction at low activation temperatures located between 200 °C and 275 °C, would improve the getter surface activity leading to high sorption performance. And secondly, the surface poisoning effect by the adsorbed contaminant species (CO, CO_2_, C–C, H–C, etc.) induces carbide phase (TiC, ZrC) formation, which reduces the metallic site density and, hence, leads to lower getter surface activity and sorption performance. 

### 3.3. Static Sorption Characterization of Non-Evaporable Getters

**Figure 5:**
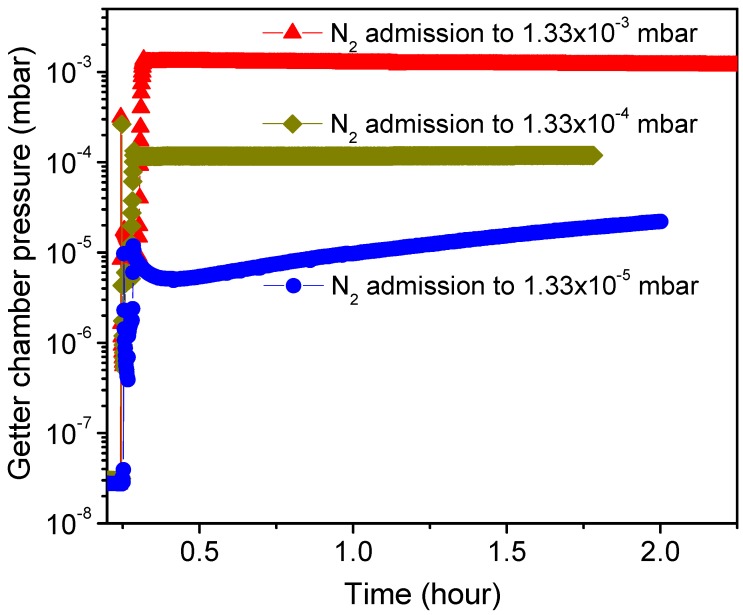
Variation of vacuum chamber pressure without the getter sample (reference test) for N_2_ gas admitted into the chamber to different pressure levels.

Since the residual gas pressure in a vacuum chamber is very sensitive to the getter surface activity, therefore to reveal the activation effect on the getter surface metallic state evolution, the time dependence of the getter chamber pressure (P–time curve) was recorded as described in Section 2. Basically, after baking-out the whole getter test system, this method measures the pressure change in the getter chamber after activating the getter, cooling it to room temperature, isolating the getter chamber from the pumping line, and then immediately admitting an amount of sorption gas to a certain pressure level into the getter chamber. However, having a reference test to which the effect of the getter on the vacuum pressure can be evidenced, the vacuum chamber characteristics without the getter were also measured. Figure 5 shows pressure–time dependency curves, with no getter incorporated in the chamber, recorded immediately after admitting N_2_ gas into the UHV sealed chamber to different pressure levels such as 1 × 10^−^^3^, 1 × 10^−^^4^ and 1 × 10^−^^5^ Torr (corresponding, respectively, to 1.33 × 10^−^^3^, 1.33 × 10^−^^4^ and 1.33 × 10^−^^5^ mbar as indicated on the plot) and closing the controller pressure leak valve (VLV). As can be seen, no significant change in the pressure was observed for the chamber sealed at higher N_2_ gas pressures of 1 × 10^−^^3^ Torr and 1 × 10^−^^4^ Torr. However, the sealed chamber with N_2_ gas admitted at 1 × 10^−^^5^ Torr showed first a slight detectable gettering effect manifested as a small decrease in pressure lasting just for few minutes (~5 min), followed then by a gas release effect manifested as a moderate pressure increase with time. Such pressure evolution is generally related to factors affecting the vacuum chamber stability like gas adsorption by the chamber walls that reduces the pressure at the beginning of chamber sealing, and walls outgassing with other chamber parts that lead later to a pressure increase when the wall sorption rate becomes less dominant. It can be deduced that the static sorption characteristics performed in a vacuum chamber admitted with N_2_ gas limited to a pressure of 1 × 10^−5^ Torr would be useful and informative for getter performance characterization. In fact, such a low pressure appeases the fast gettering saturation of the getter by lasting the sorption measurement with time, and this is practical for the analyses of the gettering process behavior. Therefore, any difference in the getter sorption characteristics to the reference test will be systemically related to the getter effect. In addition, it is worthwhile to note that the reference test pressure level and pressure evolution tendency were not changed after getter-chamber heating at different temperatures up to 450 °C; using the same annealing conditions as in the getter activation.

**Figure 6:**
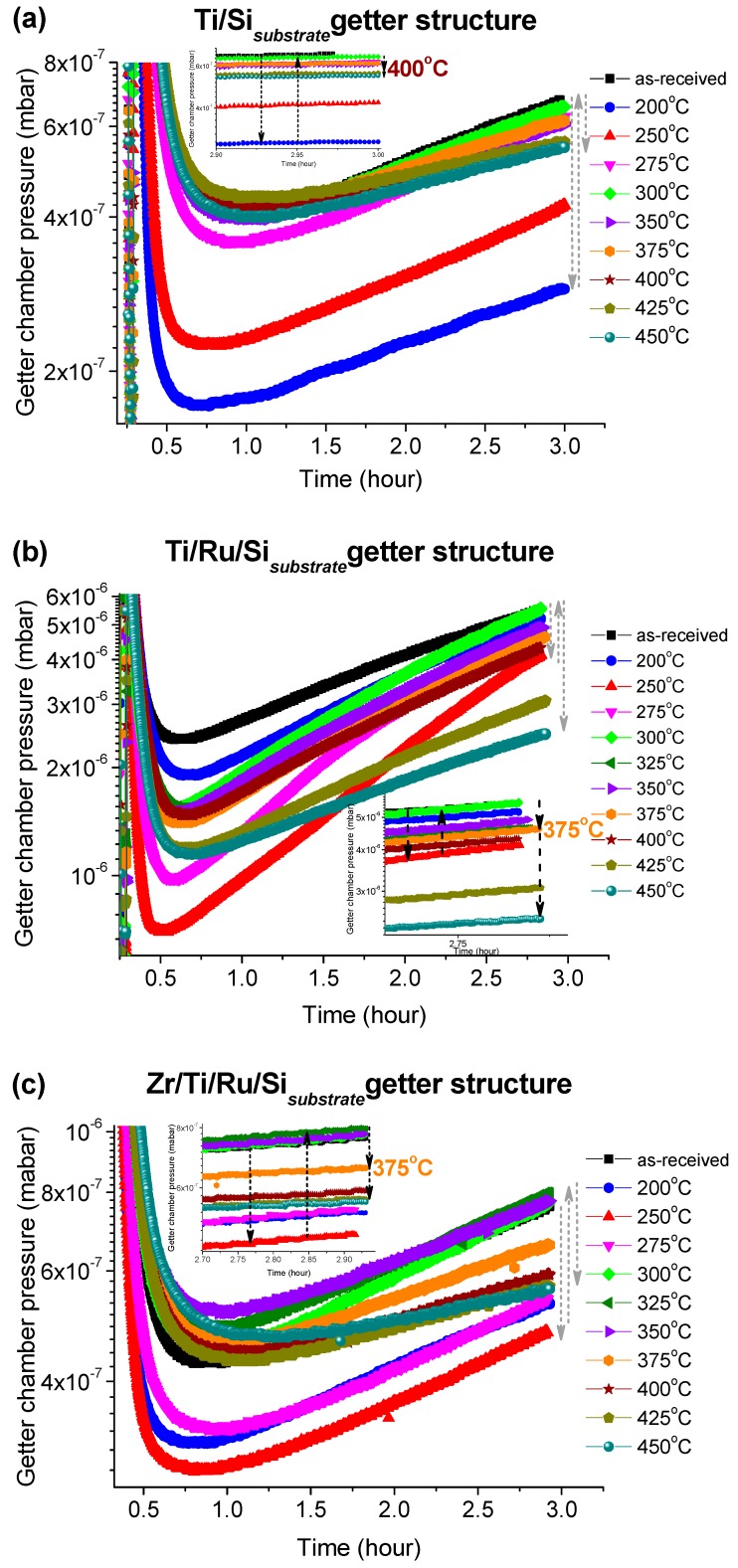
Variation of the getter chamber pressures after different activation temperatures. (**a**) Single layer Ti/Si NEG; (**b**) multilayer Ti/Ru/Si NEG and (**c**) multilayer Zr/Ti/Ru/Si NEG. Insets are detailed views of the magnified zone of pressure evolution in the right side of the P–time curves.

The pressure–versus–time curves after each activation temperature for the three investigated getters are shown in Figure 6a–c. These curves were recorded immediately after closing the leak valve (VLV) with which N_2_ gas was admitted into the UHV sealed getter chamber to a pressure of 1 × 10^−5^ Torr. It can be seen that all getters with different structures exhibit a similar N_2_ sorption trend. The curves indicate that the getter chamber pressure decreased quickly and reached a kinetic equilibrium corresponding to the minimum pressure within a period of time; varying from 0.5 to 1.5 h depending on the thermal activation temperature value. After this equilibrium, the pressure began to progressively increase with measurement time. Such behavior could be mainly attributed to the vacuum chamber walls and the sample holder outgassing, whose flow rate became higher than the rate of the gettering process. It was also seen that in the early stage of the pressure–time records as the rate of the pressure decreasing was higher as the reached equilibrium pressure level was minimal (minimum pressure value on the curve). This minimum level was reached for, both, the multilayered Ti/Ru/Si and the Zr/Ti/Ru/Si NEGs at the activation temperature of 250 °C, except for the single-layered Ti/Si NEG sample with which the minimum pressure level happened after activating at 200 °C. Above those activating temperatures, the equilibrium pressure levels increased and, again after reaching an activating temperature range around 375 °C, the pressure level slightly re-decreased, but effectively only for the multilayered getters with Ru sub-layer. Furthermore, such structured getters containing a Ru seed sub-layer (Ti/Ru/Si and Zr/Ti/Ru/Si NEGs) at higher activation temperatures (425 °C and 450 °C) manifested a notable resistance to the outgassing effect by lowering the pressure level as is seen at the right part of the curves at around 3 h of pressure recording (see Figure 6b,c).

From the getter effect on the pressure change during the thermal activation process and the temperature in situ XPS analysis of the getter surfaces, the equilibrium pressure minimum levels measured at an activation temperature of 250 °C correspond to special changes of the getter surface chemical properties as already probed by the XPS spectra at around 275 °C in our investigated samples and 250 °C in the literature [27,30,32]. For this temperature range, the change in the getter surface chemical states during the activation was demonstrated to be related to hydroxide and hydrocarbon dissociations and diffusion into the getter surface, accompanied simultaneously with oxygen bulk diffusion primed through the reduction of the passive oxide layer. Generally, this described mechanism leads to a fresh getter surface manifesting more metallic sites favorable for more N_2_ sorption and pressure lowering. However, in the subsequent activation temperature of 300 °C, the diffused carbon in the getter sub-surface and probably also the nitrogen stuck on the getter surface from the previous sorption test, react with the appeared metallic Ti^0^ (Zr^0^) elements in forming carbide and nitride compounds. Thus, leading to a lowered number of metallic sites on the getter surface that consequently weakens N_2_ sorption by manifesting a low rate of pressure decrease and a rise in the equilibrium pressure level. For a further activation temperature increase, it was seen that the pressure decrease rate continued to become slower and the equilibrium pressure level also continued to increase. This behavior is presumably due to an important increase to the poisoned metal sites (carbide/nitride) on the getter sub-surface, despite the metallic peak (Ti^0^, Zr^0^) signal increase as probed in the XPS profiles with an increasing of the activation temperature (Figure 4a). After activating at 375 °C and above, the equilibrium pressure levels and the right side of the P–time curves underwent just a little decrease indicating that all investigated getters were not completely activated as was proved by the incomplete disappearance of the XPS peaks related to the metal suboxides (Ti^x+<4^, Zr^x+<4^) and the C 1s orbital.

To get more information about the mechanisms involved in the nitrogen sorption kinetics by our investigated getters, the nitrogen sorption reaction mechanism was proposed by comparing the sorption rate characteristics with the help of Hirooka’s kinetic theory [33]. The reaction rate in this method can be expressed by the rate of gas pressure change due to the sorption:

(2) $$-\frac{dP}{dt}=k_{a}\cdot \left(P_{t}-P_{eq}\right)\;$$

where *k_a_* is the sorption rate constant, supposedly independent of pressure; *P_t_* is the getter chamber pressure at an arbitrary time; and *P_eq_* is the pressure at the final equilibrium state. 

The solution of Equation (2) allows us to extract the ka from the slope of the straight line expressed by Equation (3):

(3) $$\ln\left[\frac{\left(P_{t}-P_{eq}\right)}{\left(P_{0}-P_{eq}\right)}\right]=-k_{a}\cdot \;t\;$$

where *P_0_* is the initial pressure. 

**Figure 7:**
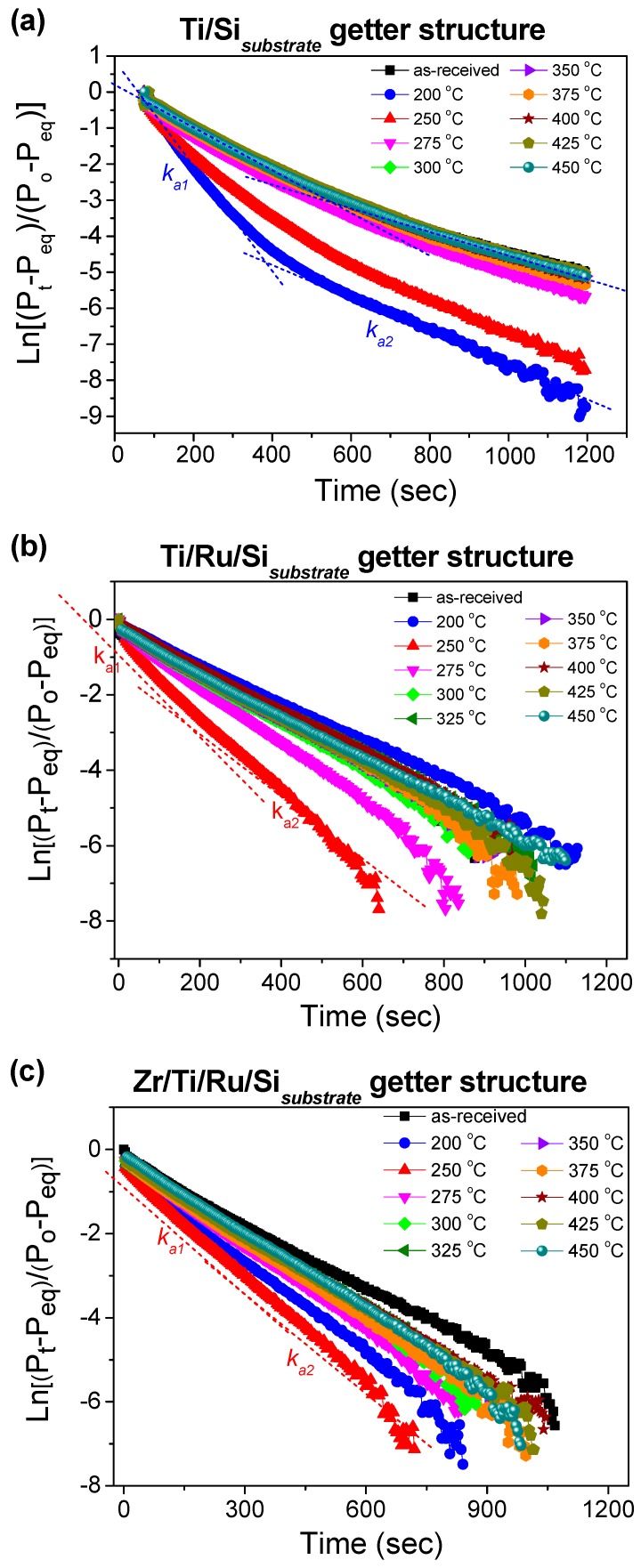
Kinetic curves for nitrogen sorption after different activation temperatures; (**a**) single layer Ti/Si NEG; (**b**) multilayer Ti/Ru/Si NEG and (**c**) multilayer Zr/Ti/Ru/Si NEG.

The typical plots of ln[(*P_t_* − *P_eq_*)/(*P*_0_ − *P_eq_*)] versus reaction time, from which the nitrogen sorption reaction rate constant characteristics (*k*_*a*1_, *k*_*a*2_) can be extracted, are depicted in Figure 7a–c. It was observed that for the Ti upper-layer based getter without a Ru sub-layer (Ti/Si NEG), the curves can fit into two different linear segments characterized by two different slopes corresponding, respectively, to a rapid sorption stage with *k*_*a*1_ constant and a stable sorption one with *k*_*a*2_ constant. Whereas, for the Ti and Zr upper-layer based getters with a Ru sub-layer (Ti/Ru/Si and Zr/Ti/Ru/Si NEGs), the curves fit into almost only one aligned linear segment including the rapid and stable sorption stages with approximately equal slopes *k*_*a*1_ ≈ *k*_*a*2_.

**Figure 8:**
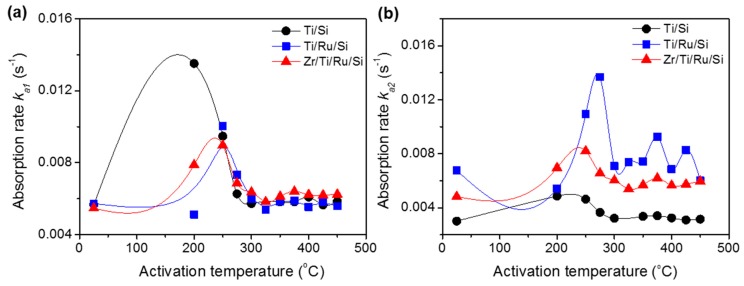
Nitrogen sorption rate constants, *k_a_*, at different activation temperatures. (**a**) Reaction rate constant, *k_a_*_1_, in the rapid sorption stage; (**b**) reaction rate constant, *k_a2_*, in the stable sorption stage.

The reaction rate constants (*k_a_*_1_, *k_a_*_2_), calculated after each activated temperature for all studied getters, are shown in Figure 8a,b. The *k_a_*_1_ constants demonstrate an increase until the 250 °C activating temperature (except Ti/Si NEG with *k_a_*_1_ max at an activation temperature of 200 °C) followed by a subsequent decrease with increasing activation temperatures. The origin of such behavior has already been discussed above in the sense that a higher rate constant was found to be related to getter surface cleaning (NEG sub-surface reduction by metal-hydroxide formation) and its drop in carbide/nitride sites formation. Regarding the rate constant *k_a_*_2_, whose effect was considerable only in the getters without a Ru sub-layer, their values did not undergo any substantial change with activation temperature. 

As the variation in the slope of the Equation (3) plot, during the nitrogen sorption, is related to the rate controlled sorption mechanism change. Consequently, for the Ti upper-layer based getter without a Ru sub-layer (Ti/Si NEG), the high reaction rate (*k_a_*_1_) could be associated with the N_2_ adsorption on the getter surface and the lower reaction rate (*k_a_*_2_) could be associated to the absorption by diffusion of N_2_ into the getter bulk, which took over to control the sorption process when the surface was saturated [34,35,36]. Whereas for the Ti and Zr upper-layer based getters with a Ru sub-layer (Ti/Ru/Si and Zr/Ti/Ru/Si NEGs), the manifestation of only one slope (*k_a_*_1_ ≈ *k_a_*_2_) in the nitrogen sorption curve predicts that the sorption process was controlled simultaneously by N_2_ adsorption and diffusion. From the above analysis and predicted hypotheses, the difference in sorption mechanisms demonstrated by the investigated getters can be assumed to be specifically due to the getter microstructure, which was engineered in a highly leafleted columnar and porous structure with a highly rough surface morphology when the getter structure comprised a Ru metal as a thin film sub-layer. Therefore, it would be expected for the getters presenting only one sorption stage in their sorption kinetic curves to show better sorption performance properties.

### 3.4. Dynamic Sorption Characterization of Non-Evaporable Getters

**Figure 9:**
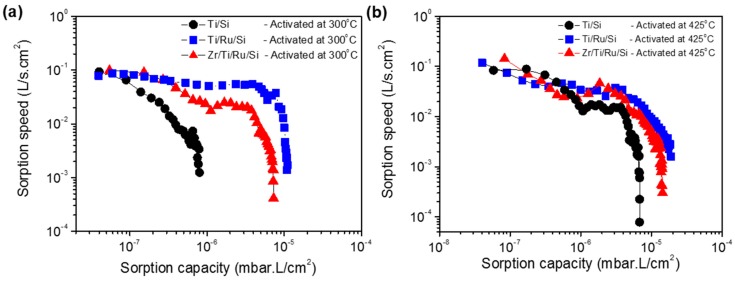
Sorption property comparisons of single-layered and multilayered NEGs. (**a**) NEGs activated at 300 °C; (**b**) NEGs activated at 425 °C.

In the following, the sorption performance characterizations of the three investigated NEGs were measured and compared. Figure 9a shows the sorption speed as a function of sorption capacity at room temperature for Ti/Si, Ti/Ru/Si and Zr/Ti/Ru/Si getters activated at a temperature of 300 °C for 1 h. It can be seen that the sorption speed for the single layer Ti/Si NEG decreases rapidly and ends with a low sorption capacity at around 1 × 10^−6^ mbar·L/cm^2^. While for the Ti/Ru/Si and Zr/Ti/Ru/Si multilayered NEGs, the sorption speeds were higher and manifest as a slightly inclined plateau shape and diminish at a sorption capacity on the order of 1 × 10^−5^ mbar·L/cm^2^. For a higher activation temperature of 425 °C (Figure 9b), the three getters exhibit together a plateau having approximately the same sorption speed evolution. However, the multilayered Ti/Ru/Si and Zr/Ti/Ru/Si NEGs have demonstrated a sorption capacity roughly four times higher than the one measured with the single layer Ti/Si NEG. The low measured sorption properties, previously exhibited by the three getters after the activation temperature of 300 °C (see Figure 9a), were directly corroborated by the diminution of the reaction rate constant (Figure 8a) measured from the nitrogen pressure–time evolution. The sorption weakening, in this activation temperature range, can be accounted for by the getter surface poisoning effect that results, during the activation process, in reacting contaminating gaseous impurities (CO, CO_2_, C–C, H–C, N_2_, etc.) adsorbed on the getter surface with getter metal. Thus, leading to metal carbide (probably also nitride) species formation on the getter surface as determined by XPS analyses. This poisoning process can spread out in a thin sorption blocking layer leading to a sorption decrease and even the degradation of the gettering ability. 

**Figure 10:**
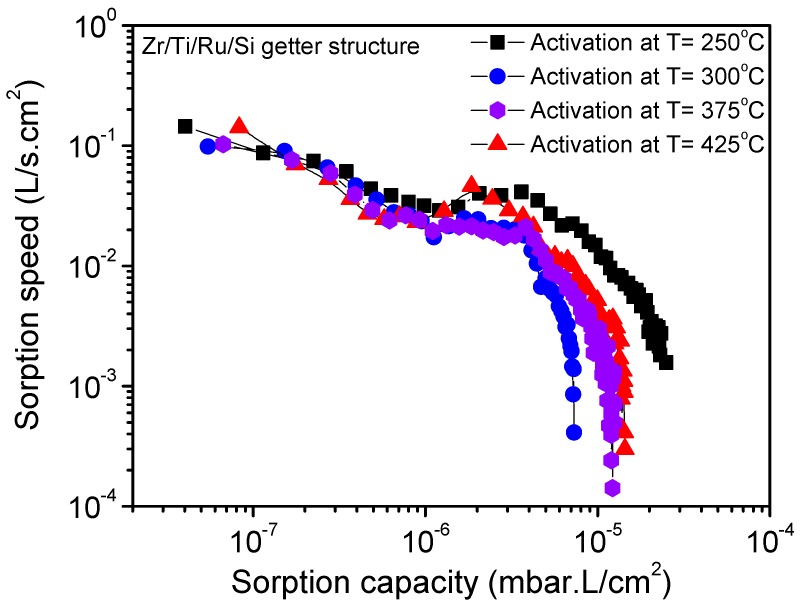
Sorption characteristics of the multilayered Zr/Ti/Ru/Si NEG after different activation temperatures for one hour.

The poisoning phenomenon that hinders the gettering activity was revealed by the two above static sorption (P–time) and XPS analyses, to be primed just after the thermal activation over 250 °C and was diminished by raising the activation to high temperatures over 350 °C. Figure 10 shows the nitrogen sorption property evolution at room temperature for the Zr/Ti/Ru/Si multilayered getter after being activated at different temperatures for 1 h. It can be seen that for the activation at 250 °C, the resultant sorption characteristic curve has shifted to a higher sorption capacity value, while the activation at a higher temperature of 300 °C made the sorption characteristic curve shift to a lower range. Furthermore, for activations at higher temperatures, 375 °C and 425 °C, the sorption capacity limit seemed to stabilize in between. The lowering of the sorption capacity for activation temperatures over 250 °C was explained above to be due to the poisoning effect. However, the pronounced sorption capacity, observed at an activation temperature of 250 °C, was effectively associated with metal-hydroxide cluster formation on the getter upper surface. The getter surface state transformation, while activating at 250 °C, was elucidated. During the activation process, the gaseous impurities (O–H and C–H groups), already adsorbed on the getter surface, dissociated and resulted in hydrogen internal diffusion. This locally diffused hydrogen reacts in the passive layer oxide by forming a distributed metal-hydroxide cluster in the getter sub-surface [30,31,32], thus inducing an important reduction of the passivation oxide layer by establishing a high coexistence of sub-oxidized and metallic states; a configuration which is very favorable for high gettering activity. The reestablishment of the sorption capacity, after activating at 375 °C and above, is due, in addition of oxide dissolution/diffusion, to decomposition and diffusion of poisoning compounds, which probably concerned only the metal-hydroxides not including carbides as the C 1s peak did not undergo any considerable decrease in this temperature range (Figure 4b). Thereby the getter surface activity was re-improved. However, in spite of the obtained better sorption properties at a low temperature of 250 °C, a question is still open whether or not to exploit the getter activation at a higher temperature of 375 °C. In fact, this temperature is also advantageous; it is related to an effective dissolution of the passivation layer compounds into the seeping microstructure, engendered in the getter-bulk by the appropriate additional Ru sub-layer in the getter structure. Furthermore, it is also included in the temperature range compatible with the MEMS sealing process.

It is to be noted that in the getter film patterning processes, the use of organic solution components can degrade the getter sorption performance or even lead to sorption failure. Given that the getter metal surface is very sensitive to any external chemical compounds coming from photoresist and solvent products used in the photoresist deposition and removal, the interaction of these elements with the getter would deteriorate the getter surface morphology stability and sorption ability. In addition, as most metals are hard to etch by dry plasma etching, the MEMS patterned getters can be performed easily using a physical shadow mask, but the patterning size of this technique is limited only in the range of the micrometer-scale. However, in the case of lateral getter film patterning, using lift-off or other standard lithography methods, a post wet cleaning step restoring the getter performance would be highly recommended. To this end, an investigational study is needed to select the appropriate cleaning solution, which can interfere with the used specific metal of the getter in order to both pre-activate the getter surface by removing the oxides and to clean the getter surface by eliminating the contaminants.

## 4. Conclusions

Non-evaporable getters structured in single and multilayered thin films such as Ti/Si, Ti/Ru/Si and Zr/Ti/Ru/Si were deposited and analyzed in terms of microstructure and morphology formation, activation mechanism, and sorption performances. The insertion of Ru as a seed sub-layer has shown, in term of microstructure growth, a significant improvement to the microstructure modification in the fine vertical leafleted texture with a rough and porous morphology of the upper getter surfaces. It was also found that NEGs with a Ru seed sub-layer exhibited lower triggering activation temperatures than unseeded ones. The temperature values extracted from the right side of P–time curves at the end of tests, just at the sudden jump after the second downward pressure level drop as seen by the broken arrows in the magnified zone in the insets of Figure 6a–c, were 400 °C for a single layer Ti/Si NEG and ~375 °C for both the layered Ti/Ru/Si and Zr/Ti/Ru/Si NEGs. These temperatures were a little higher than the temperatures related to the start of the oxide reduction as probed by the increase of metallic states (Ti^0^, Zr^0^) of $\mathrm{Ti}\;2p_{3/2}$ and $\mathrm{Zr}\;3d_{5/2}$ XPS peak intensities (Figure 4a) and a decrease of the O 1s XPS profile intensity (not shown here). Such behavior can be explained by the pressure sensitivity to the poisoning effect, which takes over by reducing the getter surface active site density. Moreover, it is to be noted that the increase of the XPS metallic signal was also influenced by the metal-carbide contribution since this one manifests almost at the same bending energy as the one of the metallic state. Hence, the consideration of activation temperature from pressure evolution measurements is more credible than from the XPS data analyses that need high skills and efficient software tools for spectral deconvolution.

For the analyses in terms of activation mechanism, from the correlation between the XPS analyses and the sorption pressure evolution, the getter activation passes through three stages; at a moderate temperature until 250 °C, a formation of metal-hydroxide clusters just under the upper getter sub-surface allows higher sub-oxide and metallic state sites creation, which results in the higher sorption rate observed in the pressure–time curves. Then at around 275 °C and up to 375 °C, the formation of a carbide species takes over, which reduces considerably the metallic sites on the getter surface. This results in lower sorption rates and an upward shift of the pressure–time curves. Finally, after 375 °C and up to 450 °C, despite the probed oxide dissolution and diffusion into the getter bulk, as confirmed by the continuous increase of the Ti^0^ and Zr^0^ peaks intensities in the Ti 2p and Zr 3d XPS profiles, the carbide species did not undergo any significant decomposition and diffusion as indicated by the slight variation of the C 1s XPS profile. Such behavior was physically manifested by a slight downward re-shift of the pressure–time curves, indicating that the getter surface was refreshed but the activation process was not completely achieved. To avoid the carbon poisoning effect, a covering of the getter upper surface by a thin protective layer of Cr, Ni, Pd or Pd–Ag alloy deposited in situ during the getter process would be of great interest for activating the getter at a lower temperature, less than the 375 °C already determined for the layered getters with Ru as a sub-layer.

In addition, from the analyses of the pressure–time characteristics plotted according to Hirooka’s model, it was demonstrated that with a Ru sub-layer in the multilayered NEG structure, the gettering mechanism was controlled simultaneously by gas adsorption and diffusion processes, while a single NEG system without a Ru sub-layer manifested a gettering behavior evolving into two sequentially steps; the gas adsorption process first, when the surface sorption is dominant, followed by the gas diffusion process when the bulk sorption became predominant with a diffusion rate lower than the adsorption rate. 

Finally, regarding the multilayered NEGs containing a Ru sub-layer, getter sorption performances were found to be directly related to the sorption rate constants determined from the static sorption-activation temperature dependence measurements. As can be concluded, the getter sorption performance can be predicted to be maximal at an activation temperature of 250 °C, feeble for temperatures located in the interval 250 °C < T < 375 °C, and satisfying for temperatures from 375 °C and above. Also, as found in this study, the modulation of the activation temperature by an appropriate sub-layer addition would allow performing in situ NEG activation while MEMS packaging with a temperature adequate and compatible for the MEMS cavity sealing process parameters.

## Figures and Tables

**Table 1:** Average leaflet width size and NEG upper surface roughness.

Getter Samples ➔	Ti/Si NEG	Ti/Ru/Si NEG	Zr/Ti/Ru/Si NEG
Leaflet Width Size	225 nm	200 nm	170 nm
Roughness (Ra)	10.089 nm	16.654 nm	8.958 nm
